# Intake Biomarkers for Nutrition and Health: Review and Discussion of Methodology Issues

**DOI:** 10.3390/metabo14050276

**Published:** 2024-05-10

**Authors:** Ross L. Prentice

**Affiliations:** Division of Public Health Sciences, Fred Hutchinson Cancer Center, Department of Biostatistics, University of Washington, 1100 Fairview Avenue North, P.O. Box 19024, Seattle, WA 98109-1024, USA; rprentic@whi.org; Tel.: +1-206-930-1884

**Keywords:** biomarker, chronic disease, dietary assessment, hazard ratio, metabolomics, mortality, blood metabolomics, regression calibration, urine metabolomics

## Abstract

Metabolomics profiles from blood, urine, or other body fluids have the potential to assess intakes of foods and nutrients objectively, thereby strengthening nutritional epidemiology research. Metabolomics platforms may include targeted components that estimate the relative concentrations for individual metabolites in a predetermined set, or global components, typically involving mass spectrometry, that estimate relative concentrations more broadly. While a specific metabolite concentration usually correlates with the intake of a single food or food group, multiple metabolites may be correlated with the intake of certain foods or with specific nutrient intakes, each of which may be expressed in absolute terms or relative to total energy intake. Here, I briefly review the progress over the past 20 years on the development and application intake biomarkers for foods/food groups, nutrients, and dietary patterns, primarily by drawing from several recent reviews. In doing so, I emphasize the criteria and study designs for candidate biomarker identification, biomarker validation, and intake biomarker application. The use of intake biomarkers for diet and chronic disease association studies is still infrequent in nutritional epidemiology research. My comments here will derive primarily from our research group’s recent contributions to the Women’s Health Initiative cohorts. I will complete the contribution by describing some opportunities to build on the collective 20 years of effort, including opportunities related to the metabolomics profiling of blood and urine specimens from human feeding studies that approximate habitual diets.

## 1. Introduction

Following some decades of having only a handful of dietary intake biomarkers, for example, for total energy [1] and total protein [2], metabolite profiling in specimens from the blood, urine, and other body fluids has come to be regarded as a potential major source for objective intake assessments for many other dietary variables. The use of metabolomics profiles in body fluids for the identification of biomarkers for foods, nutrients, or dietary patterns has been the subject of considerable research for the past two decades. Progress in using the concentrations of small molecules, with molecular weights typically below 1500 Daltons (i.e., the metabolome), for intake biomarker identification has been considerable. Several author groups have reviewed this progress [3,4,5,6,7,8]. For example, Scalbert and colleagues described metabolomics, in a 2014 review, as providing a ‘window over dietary intake’ and they noted that the food metabolome, the subset of the metabolome that derives from diet, includes more than 25,000 compounds, most of which are further metabolized in the human body, and is extraordinarily complex [3]. These authors also noted that dietary exposures have traditionally been measured using self-report methods, and that ‘a number of random and systematic errors are inherent in such methods’ [3]. This and the other cited reviews [3,4,5,6,7,8] echo and elaborate the need for objective measures of dietary intake, whether for foods, nutrients, or dietary patterns, and emphasize the substantial assessment opportunity afforded by variability in the food metabolome. For example, Brennan, Hu, and Sun in a 2021 review concluded that ‘Metabolomics has great potential in nutritional epidemiology’ and that ‘Harnessing this potential will help address some of the shortcomings of the field. Specifically, the use of food intake biomarkers can help address measurement error in self-reported dietary intake data…’ [8].

While the reader may expect a review article on ‘Intake Biomarkers for Nutrition and Health’ to summarize the detailed contributions to intake biomarker identification, validation, and applications that were previously reviewed, while bringing in additional recent contributions, I have chosen to perform otherwise for this contribution. Specifically, it seems timely to present and discuss the criteria needed for the development and application of dietary intake biomarkers, with the hope of facilitating a research agenda for yielding intake biomarkers that strongly contribute to nutritional epidemiology association studies. In doing so, I apologize to the authors whose relevant work may not be specifically cited, and I further apologize that the focus I have chosen results in a rather detailed emphasis on the work of our own Women’s Health Initiative (WHI) research group.

## 2. Dietary Intake Biomarkers: Preamble

### 2.1. Are Dietary Intake Biomarkers Strongly Needed for Reliable Nutritional Epidemiology Association Analyses?

Surprisingly, the fundamental question given above does not yet have a clear answer. For example, in their 2018 review of the potential of metabolomics-based biomarkers to improve dietary assessment, Gausch-Ferre, Bhupathiraju, and Hu [7] wrote that ‘Still, this technology is intended to be complementary, rather than a replacement, to traditional well-validated dietary assessment methods such as food frequency questionnaires that can measure usual diet, the most relevant exposure in nutritional epidemiologic studies’. If it is true that self-reported dietary data, such as food frequency questionnaire (FFQ) assessments, are ‘well-validated’ and can lead to reliable nutritional epidemiology associations universally, then the role of dietary biomarkers would evidently be limited to replication studies and studies of diet and disease mechanisms. On the other hand, if the available self-report assessments do not have such strong validity, then intake biomarkers could have a fundamental role in the investigation of primary diet and disease associations.

One can organize dietary intake at a specific time in a study participant’s lifetime into total energy (calories) intake as reflective of intake quantity, and ratios of the intake of foods and nutrients to total energy as reflective of dietary composition. Let us first consider the properties of the available dietary assessment methodologies for total energy intake.

### 2.2. Objective Measurement of Total Energy Intake

Assessment of the properties of self-reported dietary data requires objective intake measures for comparison. Using replicates of the same or another self-report assessment for this purpose is suspect, since the multiple self-report assessments may share systematic biases and therefore have correlated measurement errors. However, it is usually not sensible to expect objective measures to be available that accurately and precisely measure absolute or relative (to energy) intakes, even for short-term intakes. For example, in a controlled human feeding study, there will typically be some uncertainty concerning individual food and beverage intakes (hereafter, ‘food’ intakes). In addition, for total energy and other nutrient intakes, one expects some uncertainty in the nutrient databases that support the conversion of food intakes into nutrient intakes. Accordingly, for objective intake assessments, the best version that may be achievable for free-living individuals will incorporate a random error component.

For short-term energy expenditure (TEE), such an objective measure can be constructed via the doubly labeled water (DLW) method [1]. This impressive method involves the provision of a drink, e.g., 8 oz., that includes deuterium and oxygen-18 labeled H_2_O at the beginning of a protocol period, typically a duration of 2 weeks. Deuterium leaves the body as water (HDO), while oxygen-18 leaves the body as water plus carbon dioxide, so that the production of carbon dioxide, the end product of energy metabolism, can be accurately assessed over the protocol period. Moreover, the DLW measure of (daily) TEE provides an accurate TEE assessment in humans not only during times of weight stability, but also during times of weight loss or weight gain [9,10]. Under the assumption that the DLW-TEE measures the total (daily) energy intake over the protocol period, several research groups, including our own, have compared TEE values to the (daily) self-reported total energy intake over a similarly short time period. Some of these studies have been large enough to evaluate properties in relation to participant characteristics, e.g., [11,12,13,14,15]. These studies, in various populations using various self-report dietary tools, have found strong energy underestimation among study participants with a high body mass index (BMI) and/or body fatness. For example, in the Women’s Health Initiative (WHI) cohorts of postmenopausal US women, energy intake was evidently underestimated by 30–40% among the overweight and obese participants when using food frequency questionnaires (FFQs) for dietary assessment [14,15]. Comparable results were also obtained when using 4 day food records or three 24 h dietary recalls for the intake assessments. Furthermore, the underestimation was greater among the younger than the older postmenopausal women, and among certain racial or ethnic minority populations. This type of systematic bias, if uncorrected, thoroughly invalidates corresponding studies of the association between energy intake and clinical outcomes, implying a strong need for alternative assessment approaches. One such approach uses the TEE assessments in biomarker sub-cohorts of an overall study cohort to correct, or calibrate, self-report assessments and then relates the calibrated energy intake to disease risk outcomes. Using this approach, we estimated associations between FFQ-calibrated energy intake and major disease outcomes (i.e., total and specific cancers; total and specific cardiovascular diseases (CVDs); and type 2 diabetes) in WHI cohorts. These analyses yielded associations that were mostly strong and positive when using calibrated energy but were also mostly null if self-reported energy intake assessments were used instead [16,17,18]. A possible limitation of this approach, however, is that the calibrated energy intake depends only weakly on the self-reported energy intake and more strongly on BMI and other sources of systematic bias, raising the possibility that the positive disease associations were attributable to overweight/obesity rather than energy intake per se. Even so, the state of overweight or obesity can be expected to reflect energy overconsumption and energy imbalance over preceding years, and a long-term high energy intake may be the more fundamental chronic disease risk factor. However, analyses relating short-term energy intake to major disease outcomes would be strengthened if TEE measurements were available on a study cohort of a sufficient size and follow-up duration for direct association analyses, thereby avoiding any use of self-reported dietary data.

We have recently reported [19] on an all-cause mortality analysis of this type among 1131 WHI participants with about 14 years of post-DLW assessment follow-up. TEE was not significantly related to mortality overall, but there was a substantial age interaction, with younger (e.g., age 60) postmenopausal women having higher mortality at higher TEE, whereas the opposite was true among the older postmenopausal women (e.g., age 80). This study used TEE as a proxy for energy intake. The two will only be equivalent if the energy stores, and hence body weight, are constant over the two-week DLW protocol period. We followed the TEE paper just described with a recent paper [20] in this same ‘DLW cohort’ that uses both TEE and changes in body weight over the protocol period to define an empirical energy intake biomarker, using a regression model building approach. Prospective analyses of this biomarker in relation to mortality similarly show a strong age interaction. These analyses also show the mortality association with energy intake to be strong and positive among younger postmenopausal women who have had stable or increasing weight during the preceding decade, but the inverse was observed among the older postmenopausal women who instead had a history of weight loss. The magnitudes of these associations suggest important relationships between energy intake and health, with benefits for lower energy intake, except at older ages where the avoidance of nutritional deficiency may be a more important dietary factor for health maintenance.

Note that these are studies are only in one population of older US women, and only for total mortality. I strongly encourage further study of the associations of total energy intake with disease incidence and mortality outcomes. Since self-reported energy intake is not useable for total energy intake assessment, this further work needs to build upon objective estimates of energy intake. Our WHI research group also considered the possibility of an energy intake biomarker based on metabolomics profiles in serum and 24 h urine. However, an agnostic approach to biomarker development using high dimensional metabolite profiles (~1000, mostly targeted metabolites) did not yield a biomarker that could explain more than a few percent of the variation in energy intake in a WHI feeding study among 153 participants [21]. It follows then that DLW-TEE is required for epidemiologic studies of energy intake and health at this time, in spite of the considerable costs and complex logistics of the DLW method.

## 3. Objective Assessment of Food Intake

### 3.1. Introduction

As already noted, for self-report intake assessment methods such as FFQs to be well-validated requires objective intake measures for comparison. However, objective intake measures for the absolute or relative (to energy) intake of most foods, nutrients or dietary patterns are not yet available for use in nutritional epidemiology applications among free-living persons. One might hope that absolute intake underestimation would be proportional to that of total energy, resulting in intake density measures from self-report assessments that have little systematic bias. On the contrary, several groups have reported a greater underreporting of certain foods, especially foods high in (self-reported) fat and sweets [22,23,24,25,26,27]. Despite these concerns, Subar et al. [28], in their thoughtful defense of ‘the value of self-report dietary data’, effectively argued that self-reported dietary ratio measures are useful for some purposes, while also asserting that they ‘do not use self-reported energy as a measure of true energy intake’. For density measures, these authors noted that underreporting may be small for some nutrients, such as protein and potassium. They also indicated, more generally, that the influences of systematic assessment biases on nutritional epidemiology association estimates may be mitigated to some extent by using other self-report assessments as reference tools for statistical correction procedures that address measurement error. For example, food records or dietary recalls could be considered as reference instruments for FFQs [29]. However, at least one such assessment instrument that avoids systematic bias is needed for this approach to yield disease association analyses without bias from FFQ systematic error. This is certainly not the case for total energy intake if food records or 24 h recalls are used as a reference instrument [15]. For some nutrient density variables, self-reported dietary data may turn out to incorporate only a little systematic bias; however, an assumption that systematic bias can be ignored for related disease association estimation would seem ill-advised at this time, unless supported in advance by objective dietary intake data.

Intake biomarkers based on metabolomic measurements have the potential to lead to the required objective measures for many dietary variables. To date, most of the published work on intake biomarker identification targets absolute intakes of foods or food groups. This seems paradoxical since densities relative to total energy may be the more important target for biomarker development for nutritional epidemiology purposes. For example, prominent dietary pattern scores are based exclusively on densities for food groups and nutrients. For instance, the Healthy Eating Index 2010 [30] is calculated by combining points related to the intake of total fruit, whole fruit, total vegetables, greens and beans, whole grains, dairy, total protein foods, seafood and plant proteins, polyunsaturated fatty acids, monounsaturated fatty acids, saturated fatty acids, refined grains, sodium, and ‘empty calories (solid fats, alcohol, added sugars)’, each relative to total calories.

### 3.2. Metabolomics Biomarkers for Foods and Food Groups: Two-Step Development Process

Much of the dietary biomarker development literature for food intake involves a two-step process. The first step aims to identify candidate biomarkers for a list of foods or food groups, while the second aims to establish biomarker validity through small-scale human feeding studies involving varying levels of foods under study.

Gonzalez-Pena and Brennan [7] provide a nice introduction to the metabolomics platforms used in the search for, and in the application of, metabolomics-based (absolute) intake biomarkers. These include nuclear magnetic resonance (NMR) spectrometry platforms having ‘high reproducibility’ and mass spectrometry (MS)-based platforms having ‘great sensitivity’. In either case, the approach may be targeted to a specified set of metabolites, or untargeted with the potential to assess hundreds or thousands of metabolites. Metabolite concentrations in the samples processed, relative to that for the average for other measured small molecules, are typically recorded. These authors [7] also cite several detailed reviews of metabolomics platforms used in this research.

Most of the extensive related literature is devoted to the first step in the biomarker identification process, and typically focuses on the estimation of the correlations between metabolite measurements from blood or urine with corresponding measures of food/food group intakes, and with due account for multiple testing. This work has generated hundreds of potential biomarkers for a wide range of foods and beverages. For example, Gonzales-Pena and Brennan [7], in their 2019 review of metabolomics-based advances for nutrition and health, provided a list of reports supporting one or more candidate biomarkers. These included 13 such reports proposing candidates for vegetables, 19 for fruits, 2 for legumes, 2 for soy products, 5 for grains, 15 for meats, 5 for dairy products, 19 for beverages, 4 for cocoa/chocolate, and 4 for nuts, that collectively proposed several hundred candidate intake biomarkers for foods or food groups. Much of this work, as well as more recent related work, has taken place in sub-studies with a moderate number of participants (e.g., a few hundred) in large epidemiologic cohorts. Several such reports have arisen from FoodBall, a collaboration among 13 primarily European countries [31]. Studies drawing participants from several other, primarily American, cohort studies, e.g., [4,32,33,34,35] have also contributed to this effort. In fact, the candidate biomarker identification research has exploded to such a degree that Gao and colleagues [36] have found it useful to propose a special classification scheme for dietary and health biomarkers, and Pratico et al. [37] proposed guidelines for the review of potential biomarkers for specific foods, including both metabolomic and non-metabolomic candidates.

Nearly all of these reports use some form of self-reported dietary data as the comparator to identify metabolomics correlates of food intake. This seems surprising as a research strategy since the need for intake biomarkers in nutritional epidemiology arises primarily from uncertainty concerning the magnitude of random and systematic measurement errors in self-reported dietary data. Accordingly, there is an associated strong need to validate all such candidate biomarkers for them to become established as providing objective assessments of food intake.

The second step in the biomarker development process aims to filter candidate biomarkers to identify those meeting pertinent validation criteria. Dragsted et al. [38] have proposed eight criteria for dietary intake biomarker validation, namely plausibility, dose–response, time–response, robustness, reliability, stability, analytical performance, and inter-laboratory reproducibility. The first criterion (plausibility) focuses on biomarker specificity, with attention given to whether the putative biomarkers are derived from the food/food group under study, either directly or following metabolism. The second (dose–response) is concerned with sensitivity and examines the quantitative biomarker response to differing sources and differing intake levels of study foods. The third (time–response) deals with the temporal relationship between food intake and candidate biomarker concentrations and may influence the type of specimen needed for useful biomarker identification. The fourth (robustness) concerns the suitability of the biomarker among free-living persons in study populations, and the authors recommended the conduct of a controlled feeding study using habitual diet to evaluate satisfaction of this criterion. The fifth (reliability) asserts the need for high-quality reference intakes as a key component of validation. The other three criteria (stability, analytical performance, inter-laboratory reproducibility) are, respectively, concerned with the collection, processing, and storage of specimens, the quality of the metabolomics platforms, and the agreement between laboratories in biomarker assessments.

Studies aiming to validate candidate biomarkers have mostly involved human feeding studies of a modest size using a small number of levels of the food group or dietary pattern of interest. Gonzales-Pena and Brennan [7] summarized the results of 46 nutrition intervention studies ranging in size from 10 to 300, among healthy or diseased persons over various life stages, and that applied targeted or untargeted metabolomics. While not all of these aimed to validate candidate metabolomics biomarkers for specific food groups, the authors summarize that few metabolomics biomarkers had been established by this work by the date of their review. They list tartaric acid for grape intake and betaine proline for citrus intake as examples of the potential of urine-based metabolomics markers to estimate food intake, while expressing enthusiasm for the likelihood of many further developments.

### 3.3. Metabolomic Biomarkers for Foods and Food Groups: Human Feeding Study Using Habitual Diets

Human feeding studies where participants are provided an approximation to their habitual diets have some advantages for biomarker development, and potentially also for nutritional epidemiology application. As already noted, studies of this type are recommended by Dragstad et al. [38] for establishing biomarker robustness, and they can also provide the needed quality reference measures for the biomarker reliability assessment component of biomarker validation. Our research group conducted such a study [39] among 153 WHI participants in the Seattle area from 2011 to 2014. We assessed the usual diets by starting with 4 days of food records, with further evaluation through participant interviews by a nutritionist, and with adjustments for known food record biases. The resulting intakes provided a key input for the development of an individualized 3 day rotating menu over a two-week intervention period, over which DLW assessments of total energy expenditure were conducted. Participants were free-living and came to our facility for meals to be eaten on-site and to take home for later use. To support the study of nutrient biomarkers, we chose foods that had well-characterized nutrient contents to a practical extent. Departures from the provided intakes were self-reported and feeding study intakes were adjusted accordingly. These intake values provided reference intake data for biomarker development. Most of our reported biomarker development activities to date have focused on nutrient intakes, and they use metabolomic profiles in serum and 24 h urine as developed in the Northwest Metabolomics Laboratory, headed by collaborator Dr. Dan Raftery.

As an exception to the nutrient focus, the recent report by Playdon et al. [35] from this study focuses on foods and food groups using the Metabolon liquid chromatography/tandem mass spectrometry platform as applied to serum and 24 h urine specimens. Biomarker identification was considered for 56 food groups derived from the USDA Guidelines for Americans food groups, based on 1293 relative metabolite concentrations in urine and 1113 in serum. Data analysis of the log-transformed intakes and the log-transformed metabolite concentrations identified Bonferroni-corrected significant correlations for 23 foods (including beverages) and dietary supplements involving 171 distinct metabolites. For foods, estimated correlations > 0.6 resulted for citrus, broccoli, and dairy, while estimated correlations > 0.5 also for avocado, fish, garlic, grains, onion, and poultry. Importantly, correlations for the 15 highest ranked food groups were markedly higher (difference of 0.27 on average) than the corresponding correlations from the combination of four other U.S. cohorts [32,33,34,35] also using the Metabolon platform, but with self-reported dietary data as comparators. These recent analyses suggest that urine and serum metabolomics may be rather universally applicable for biomarker identification and also suggest that the use of self-reported intakes as comparators for the identification of candidate biomarkers may be quite inefficient.

### 3.4. Metabolomics Biomarkers for Nutrients and Nutrient Densities

Even though there are many epidemiologic studies reporting associations between nutrient intakes and chronic disease using self-reported dietary data, there is little literature on the development or application of metabolomics-based nutrient intake biomarkers. Much of the nutritional epidemiology literature over the past 50 years has focused on nutrient densities, especially for macronutrients and their components, for which observed associations can be viewed as complementary to those for total energy intake. For example, Willett [40], p. 269 wrote that ‘…from an individual or population standpoint, nutrient intake in relation to total caloric intake (i.e., the composition of diet) is most relevant. For this reason, nutrient intakes adjusted for total energy intake, rather than absolute nutrient intakes, are of primary interest in relation to disease risk in epidemiologic studies’. A feeding study with habitual diets, as described above, provides a context for the study of biomarker-based nutrient intakes in relation to chronic disease risk, for both absolute intakes and for intakes relative to total energy.

Before further describing the recent work by our WHI research group, it is instructive to comment on the possibility that this study design can simultaneously lead to candidate biomarker identification as well as useful validation assessments for candidate biomarkers. To amplify on this point, I will introduce some measurement error modeling considerations as follows: a principal goal of a feeding study with habitual (short-term) dietary intakes is to yield feeding study intakes that satisfy the following:

log-actual intake = log-feeding study intake + error1

where the presumably small error1 is independent of the feeding study intake and is independent of participant characteristics or exposures that may be risk factors for the clinical outcome under study. This modeling assumption will be quite plausible in well-conducted human feeding studies within the targeted study population. For nutritional epidemiology purposes, one may seek intake biomarkers for which:

log-feeding study intake = log-biomarker intake + error2         A

where error2 is independent of the biomarker intake and is independent of participant characteristics that may be risk factors for the clinical outcome under study. These equations combine to give the following:

log-actual intake = log-biomarker intake + error         B

where error = error1 + error2 is independent of the biomarker intake and of risk factors for the study outcome. Now, consider B in conjunction with a (Cox) hazard ratio model [41] of the form:

exp (zb + wa)         C

where z is log-actual intake, w is a vector of potential confounding factors, and ‘b’ and ‘a’ are corresponding hazard ratio parameters to be estimated, with primary interest in the estimation of ‘b’. Under A–C and a rare disease assumption, the parameter b can be estimated for rare outcomes simply by replacing z in C by log-biomarker intake [42], with the ‘noise’ (error) component of B, only reducing the precision of estimates of the association between actual intake and hazard ratio for the study outcome [43,44].

In the metabolomics-based biomarker development for foods, overviewed above, e.g., [35], candidate metabolite biomarkers were mostly correlated with a single food group, though specific food groups often had multiple corresponding candidate metabolites. For nutrients, such as macronutrients or their components there may be a lengthy list of foods contributing to intake, with a corresponding potentially large number of related metabolites. Linear regression of the feeding study intake on metabolites provides a convenient means for the identification of potential biomarkers adhering to A. Such an approach, regressing feeding study (*n* = 153) log-intakes on log-metabolite concentrations and participant characteristics, was used by our group [45] to propose candidate (multi-metabolite) biomarkers for total protein and total carbohydrate and their densities. The linear regression analysis has the potential to yield a candidate metabolite-based biomarker with an error term that is uncorrelated with biomarker intake and with modeled participant characteristics, thereby adhering to A under normality assumptions (which may be facilitated by these log transformations). The analysis in [45] used targeted LC-MS/MS in serum with 819 metabolites (including 664 lipids), and both NMR spectroscopy (57 metabolites), and GC-MS (275 metabolites) in 24 h urine, with all metabolites considered for inclusion in biomarker model building.

Because of the dimensionality of the metabolites, a LASSO procedure [46] was used for metabolite selection, and cross-validation was used to reduce overfitting in assessing (log-transformed) biomarker correlations with log-feeding study intakes. To avoid confounding bias in associated nutrient intake and disease risk analyses, there is an obligation to consider whether disease risk factors need to be included for the biomarker specifications to adhere to A. Biomarker development exercises of this type are reported in [45]. The proposed biomarker equations each involve several metabolites, with DLW assessments of total energy and urinary nitrogen assessments of total protein contributing strongly to proposed biomarker equations for absolute carbohydrate and protein intake, respectively, but not to proposed carbohydrate and protein density biomarkers. The resulting biomarker equations give biomarker values in other WHI biomarker studies that were used [45] to calibrate the corresponding FFQ assessments for measurement errors. The calibrated intakes were then related to incidence rates for cancers, cardiovascular diseases, and diabetes in the WHI cohorts. Several strong associations were identified with HRs considerably further from the null than the corresponding HRs were, based on FFQ data without measurement error correction [45]. Subsequent reports used this same metabolite-based biomarker development and application process for major carbohydrate and protein components [47], for total fat intake defined as total energy minus carbohydrate, protein, and alcohol energy [48], and for major fatty acids categories [49], with multiple strong disease associations reported using biomarker-calibrated intakes, for both absolute intakes and for nutrient densities.

The suitability of the intake biomarkers used in this work, developed using linear regression (with model form A considered for log-transformed feeding study intakes), can be viewed in relation to the Dragsted et al. [38] validation criteria mentioned above. The study of habitual diets in the study population of interest with its various characteristics supports the satisfaction of the robustness criterion. The use of quality feeding study intakes to approximate habitual diets attends to the reliability criterion. The metabolites appearing in proposed biomarker equations derive from specimens obtained during the feeding period, supporting the time–response requirement. Stability of the stored specimens and analytic performance of the metabolomic profiles have been documented using various quality control activities, e.g., [21]. An initial study of inter-laboratory reproducibility is underway comparing Raftery and Metabolon metabolomics profiles in serum and 24 h urine. This leaves the plausibility (specificity) and dose–response (sensitivity) criteria for consideration.

A major requirement in our proposals of metabolite-based biomarker equations for nutrient intakes, from the linear regression of log-feeding study intakes on log-metabolite concentrations, is a cross-validated percentage of feeding study log-intake variation explained (R^2^) of at least 36% (cross validated correlation of at least 60%). It is natural to seek a biomarker so that error2 in A is small, compared to variation in the potential biomarker. Variations in log-feeding study intake that are not reflected in the biomarker equation defined by the regression analysis give error2 values that depend on feeding study intake and hence, lead to a lack of biomarker sensitivity, while variations in biomarker equation values that do not correspond to feeding study intake variations imply a noisy biomarker and lack of specificity. Our arguments for plausibility and dose–response in the macronutrient papers mentioned above considered these issues only informally.

Some of the metabolites in the biomarker equations just alluded to were plausible through known food chemistry, in support of specificity. However, with high-dimensional data, there may be multiple metabolite combinations having similar R^2^-values. The regression modeling provides dose–response information toward establishing sensitivity, but complex nutritional variables may require a large number of metabolites in biomarker equations to be fully sensitive. Consider dietary fat density to illustrate this last point: An agnostic approach to biomarker regression model building did not come close to yielding a biomarker meeting a 36% R^2^ criterion in the WHI feeding study [21]. This occurs, presumably, because many more metabolites than were selected by LASSO model fitting would be needed for adequate sensitivity to a broad range of fatty acid intakes. In contrast, writing total fat density as one minus carbohydrate, protein, and alcohol densities gave biomarker values explaining much more of the feeding study fat density variation [48]. Our R^2^ criterion could be met using an agnostic biomarker development process for the more specific intakes of saturated, monounsaturated, and poly-unsaturated fat density components of total fat density [49]. However, as explained in the Discussion section of that paper, there is reason to be concerned that the related biomarkers may still lack needed sensitivity for reliable disease association analyses, presumably again because of the limited number of metabolites selected for proposed biomarkers. Additional analyses are underway using even more specific fatty acids intake classes to pursue this topic further. More generally, however, a feeding study design with habitual diets has the potential for both the identification and the nutritional epidemiology application of intake biomarkers for various foods and nutrients, both for absolute and for relative intakes.

### 3.5. Metabolomics Biomarkers for Dietary Patterns

There has been comparatively little research to date on the development and use of metabolite-based biomarkers for dietary patterns—quite an important nutritional epidemiology research area. A noteworthy exception is the study by Playdon et al. [50] showing correlations between diet quality scores based on self-reported diets and serum metabolite profiles (1316 serum metabolites) among 1336 participants in the ATBC Cancer Prevention Study cohort of Finnish smokers, for each of four healthy diet scores (Healthy Eating Index 2010; the alternative Mediterranean Diet, WHO Healthy Diet Indicator, and Baltic Sea Diet). Correlations, however, tended to be small (<0.3 in absolute value). Another interesting exception is provided by Garcia-Perez et al. [51] who used a 4 period randomized cross-over design (*n* = 26) to show that diets having contrasting WHO Healthy Eating Guideline scores could be distinguished based on high-dimensional plasma NMR spectroscopy metabolite profiles. Similarly, a cross-over trial by Esko et al. [52] showed that three dietary patterns having different macronutrient compositions could be clearly distinguished based on 333 plasma metabolites. These authors note that metabolite profiles can provide useful markers additionally for intervention adherence assessment. These papers collectively show the potential of metabolomics assessments for the objective assessment of dietary pattern scores, but research to replace self-reported diet quality scores by objectively measured metabolite combinations is still at an early stage of development.

## 4. Review of the Application of Intake Biomarkers in Clinical Outcome Association Studies

It is still early days for the application of intake biomarkers for dietary exposure assessment in nutritional epidemiology disease association studies. With intake biomarkers defined, that satisfy the criteria of Dragsted et al. [38], and that have a strong correlation with provided intake in relevant feeding study contexts, one can consider applications that measure the intake biomarkers for each participant in a cohort having sufficient size and follow-up duration for precise disease association estimation. We illustrated this approach above for the study of mortality associations with (short-term) total energy intake [20]. For another WHI application, consider the association between the carotenoid and tocopherol intakes, with related biomarkers built from serum micronutrient concentrations using the WHI feeding study mentioned above [39]. The resulting biomarker equations [53] were used to estimate (absolute) intake of these micronutrients in a sub-cohort of 5488 WHI enrollees where serum concentrations had been used for cohort dietary monitoring. Several chronic disease associations between the biomarker-assessed intakes and disease incidence were estimated; for example, lower risks of cardiovascular disease, breast cancer, and diabetes were estimated with higher intakes of alpha and beta carotene.

Studies using metabolomics intake biomarkers from stored specimens can use a nested case-control rather than cohort study design to study disease associations with much reduced costs for assembling the needed metabolomics data. For example, Playdon et al. [54] considered serum metabolites proposed as candidate intake biomarkers for various foods and contrasted these 113 potentially diet-related metabolites concentrations between breast cancer cases and controls. While there were only three significant associations for overall breast cancer, there were 19 for the estrogen-receptor positive breast cancers. Also, metabolite panels mentioned above for discrimination among healthy eating patterns were studied [50,51] and found to be associated with disease risk in the hypothesized manner.

Another approach to using intake biomarkers for nutritional epidemiology association studies uses biomarkers to adjust self-reported intakes for measurement error in cohort sub-studies of moderate size, and then use resulting calibration equations to calculate ‘calibrated intakes’ in the larger epidemiologic cohorts. This approach was considered for carotenoid and tocopherol intakes in large WHI cohorts. The results were compared with those based on the smaller sub-cohort (*n* = 5488) with measured serum micronutrient concentrations [55]. While there was considerable commonality in the two sets of results for cardiovascular diseases, cancers, and diabetes, the correspondence was less than perfect, motivating further study of the properties of the two approaches, especially for the approach involving calibration of self-reported dietary data. Also, D’Angelo et al. [56] posited an alternate approach to calibration equation development, with application to a urinary proline betaine biomarker for citrus intake.

As mentioned above, our research group has used a regression calibration approach in reports of macronutrient and chronic disease associations [45,47,48,49] with many estimated associations much stronger than corresponding associations based on self-reported dietary data, as well as in a chronic disease association study for red and processed meat [57]. In these reports, intake biomarkers relied primarily on metabolite concentrations in serum and 24 h urine. The biomarker-calibrated approach is well motivated since a calibration approach, if well substantiated, can be carried out for a broad range of dietary variables including foods, nutrients, and dietary patterns, based on a feeding study of moderate size (*n* = 153 in WHI) using habitual diets in conjunction with a biomarker sub-study of moderate size (*n* = 450 in WHI) having metabolite profiles. However, additional work is needed to more explicitly develop criteria to ensure adequate sensitivity and specificity in biomarker development with high-dimensional metabolite data, and to develop criteria more explicitly for calibration equations, including criteria for contributions to calibration equations from self-reported dietary measures, to ensure reliable corresponding disease association results.

## 5. Summary and Conclusions

Findings and perspectives from this review will be summarized in three tables. Table 1 summarizes and compares some properties for two study designs for candidate biomarker identification for foods, nutrients, or dietary pattern scores. Since biomarker studies within the established well-characterized epidemiologic cohorts have relevance for the cohort target population, we restricted the attention to cohort subsample designs that either use self-reported diet for comparator, or that use an embedded feeding study with diets that aim to approximate habitual diets. Both study designs have the potential for plausibility/specificity by examining the chemistry related to metabolites that surface as individual candidate intake biomarkers, or as one of multiple metabolites that combine to define candidate intake biomarkers. Similarly, both designs have the potential to evaluate dose–response and sensitivity to various sources, and quantities of the dietary variable under study, in an observational fashion. The dietary comparator data typically cover a few weeks or months with the first design, whereas the embedded feeding study may provide food and drink for only days or a few weeks. Repeated study design applications over time could identify candidate biomarkers for long-term intake, with either design. By carrying out candidate biomarker development in representative cohort subsamples, either study design can deliver results that are robust for the target population. The design using self-reported diet as the comparator has an uncertain reliability due to measurement errors in the dietary assessments. In contrast, the use of recorded food intakes in the habitual diets feeding study design yields good reliability. Accordingly, even though feeding studies with habitual diets may be considerably more expensive for a given study size, this cost may be more than offset by the higher correlations of metabolites with actual intake and streamlined biomarker validation procedures, e.g., [54]. A strong argument in favor of the efficient habitual diet feeding study design for intake biomarker development is its potential for valuable progress toward assessing candidate biomarker validity within the same study context.

Table 2 gives an assessment of the merits of a small-scale randomized trial design in a convenience sample of participants, as well as of a cohort subsample habitual diet feeding study design, for examining the potential biomarker satisfaction of validation study requirements. Much of the focus of candidate biomarker identification has considered the certainty and magnitude of correlation between candidate biomarkers and their dietary intake comparators, sometimes following log-transformations of both variables. A randomized trial of sufficient size with varying levels of a dietary variable of interest should have good potential to evaluate plausibility and dose–response, with good reliability, and to elucidate a strong relationship between intake and biomarker, for a well-selected candidate biomarker. Unless it is large and of a complex design, however, such a trial likely lacks robustness relative to any larger target population. The habitual diet feeding study, on the other hand, also has the potential for a strong correlation (e.g., >0.6) between the provided intake and a well-selected candidate biomarker, as demonstrated in recent applications in the WHI cohorts. With sufficient effort, this design also has a good potential to assess plausibility and dose–response, though an observational rather than a randomized fashion, while also being able to assess candidate biomarkers with robustness and reliability. Both study designs are likely to be expensive if they include sample sizes in the >100 range, but the habitual diet feeding study has evidently enhanced efficiency since it can be used for candidate biomarker identification and validity assessment for a range of dietary variables, rather than just for the dietary variable used to define groups to be compared in the randomized trial setting.

Table 3 summarizes the merits for two study designs for relating a validated intake biomarker to clinical outcomes. As noted above, biomarker–based dietary pattern score categories have shown the ability to distinguish groups with differing risk for cardiovascular and other chronic diseases (e.g., [52,53]). For disease association studies in a large epidemiologic cohort, one can consider the direct use of a validated biomarker in a sub-cohort of sufficient size and duration for precise association estimation. Or one can consider a two-stage procedure that uses the biomarker to estimate a calibration equation for dietary variables of interest by adjusting self-report intake estimates (e.g., FFQs) for measurement error in biomarker sub-studies of moderate size, and then relate calibrated intakes to disease risk in the larger cohorts having the self-reported data. The latter approach is cost-efficient in that validated biomarker values are needed only in sub-studies of moderate size, but an additional set of measurement error modeling criteria need to be satisfied for the calibrated intake estimate, and these require the self-report to explain a substantial fraction of the biomarker variation in the sub-study. In the absence of criteria like those of Dragsted et al. [38], the resulting disease association estimates will incorporate uncertainty beyond that for the direct biomarker application design. The direct application design may be horribly expensive, however, for large cohorts, and a nested case-control study approach may be needed to control biomarker assessment costs. As discussed above, however, a biomarker approach to total energy intake is presently not available using stored specimens, but metabolomics-based biomarkers for dietary density variables considered to date [45,47,48,49] do not depend materially on the DLW-based assessment of total energy intake, so energy intake may not be crucial for the conduct of case-control studies of dietary composition variables in relation to specific clinical outcomes.

In view of these summaries, I strongly recommend the conduct of further sizeable habitual diet feeding studies in other target populations, to facilitate the harnessing of the enormous potential of metabolomics in nutritional epidemiology [8], for foods, nutrients, and dietary patterns, for both absolute and relative (to energy) intakes. Intake biomarker developments using this, or other, study designs may also have valuable applications in assessing adherence to dietary interventions in randomized trials. The research summarized here has mostly focused on short-term dietary intakes, as befits the current stage of development of this large research area. However, biomarkers will be needed that appropriately assess intakes over more lengthy segments of the lifespan, with serial feeding studies having metabolomics assessments offering a viable approach. And finally, in addition to their potential to strengthen the findings of primary disease associations, metabolite profiling has the potential to provide valuable insights into the mechanisms underlying observed disease associations, especially through interactions with genotype and with the gut microbiome, e.g., [58,59,60,61]. Although it is important and interesting, this topic is beyond the scope of the current review.

## Figures and Tables

**Table 1:** Qualitative assessment of candidate intake biomarkers identified under two study designs.

Candidate Biomarker Criterion	Ability to Satisfy Criterion Study Design	
	Cohort subsample, self-reported dietary data	Cohort subsample, feeding study, habitual diet
Correlation > 0.6 with comparator	Modest	Good
Dragsted et al. [38] validation criteria:		
Plausibility (specificity)	Good potential	Good potential
Dose–response (sensitivity)	Good potential	Good potential
Time–response (dietary data time period)	Good (weeks/months)	Good (days/weeks)
Robustness (relevance to target population)	Good	Good
Reliability (quality of comparator)	Uncertain	Good
Acceptable study cost	Good	Uncertain
Acceptable study efficiency	Uncertain	Good

Candidate intake biomarker may be for food/food groups, for nutrients, or for calculation of dietary pattern scores.

**Table 2:** Qualitative assessment of ability of candidate biomarkers to satisfy validation criteria under two study designs.

Biomarker Validation Criterion	Ability to Satisfy Criterion Study Design	
	Randomized feeding trial, convenience sample	Cohort subsample, feeding study, habitual diet
Correlation > 0.6 with provided intake	Uncertain	Good
Dragsted et al. [38] validation criteria:		
Plausibility (specificity)	Good	Good potential
Dose–response (sensitivity)	Good	Good potential
Time–response (time period for provided diet)	Good (days/weeks)	Good (days/weeks)
Robustness (relevance to target population)	Poor	Good
Reliability (quality of comparator)	Good	Good
Acceptable study cost	Uncertain	Uncertain
Acceptable study efficiency	Uncertain	Good

Intake biomarker under evaluation may be for food/food groups, for nutrients, or for calculation of dietary pattern scores.

**Table 3:** Qualitative assessment of the ability to satisfy the requirements for Reliable Nutritional Epidemiology Disease Association Results, using intake biomarkers satisfying the Dragsted et al. [38] Validation Criteria under two Study Designs.

Disease Association Requirements	Ability to Satisfy Requirements of Study Design	
	Cohort study using biomarker-calibrated dietary self-reports	Cohort study with direct use of biomarker intakes
Satisfy associated measurement error modeling assumptions	Uncertain	Good
Acceptable study cost	Good	Uncertain
Acceptable study efficiency	Good	Good
Reliable disease association estimates assuming adequate confounding control	Uncertain	Good

Dietary biomarkers may be for food/food groups, for nutrients, or for calculation of dietary pattern scores.

## Data Availability

Data, codebook, and analytic code used in this report may be accessed in a collaborative mode as described on the Women’s Health Initiative website (www.whi.org).

## Abbreviations

ATBC: alpha tocopherol beta carotene; BMI, body mass index; CVD, cardiovascular disease; DLW, doubly labeled water; FFQ, food frequency questionnaire; FoodBall, Food Biomarker Alliance; MS, mass spectrometry; NMR, nuclear magnetic resonance; USDA, United States Department of Agriculture; WHI, Women’s Health Initiative; WHO, World Health Organization.
